# Monophosphoryl lipid A alleviated radiation‐induced testicular injury through TLR4‐dependent exosomes

**DOI:** 10.1111/jcmm.14978

**Published:** 2020-03-05

**Authors:** Zhe Liu, Kun Cao, Zebin Liao, Yuanyuan Chen, Xiao Lei, Qun Wei, Cong Liu, Xuejun Sun, Yanyong Yang, Jianming Cai, Fu Gao

**Affiliations:** ^1^ Department of Radiation Medicine Faculty of Naval Medicine Second Military Medical University Shanghai China; ^2^ Department of Naval Aeromedicine Faculty of Naval Medicine Second Military Medical University Shanghai China; ^3^ Department of Surgical Oncology Sir Run Run Shaw Hospital Zhejiang University School of Medicine Hangzhou China

**Keywords:** exosome, MPLA, radioprotection, testis

## Abstract

Radiation protection on male testis is an important task for ionizing radiation‐related workers or people who receive radiotherapy for tumours near the testicle. In recent years, Toll‐like receptors (TLRs), especially TLR4, have been widely studied as a radiation protection target. In this study, we detected that a low‐toxicity TLR4 agonist monophosphoryl lipid A (MPLA) produced obvious radiation protection effects on mice testis. We found that MPLA effectively alleviated testis structure damage and cell apoptosis induced by ionizing radiation (IR). However, as the expression abundance differs a lot in distinct cells and tissues, MPLA seemed not to directly activate TLR4 singling pathway in mice testis. Here, we demonstrated a brand new mechanism for MPLA producing radiation protection effects on testis. We observed a significant activation of TLR4 pathway in macrophages after MPLA stimulation and identified significant changes in macrophage‐derived exosomes protein expression. We proved that after MPLA treatment, macrophage‐derived exosomes played an important role in testis radiation protection, and specially, G‐CSF and MIP‐2 in exosomes are the core molecules in this protection effect.

## 1 INTRODUCTION

Radiation therapy is a widely used method for many human malignancies, including lung cancer, prostate cancer and colon cancer.^1^, ^2^, ^3^ However, side effects of ionizing radiation cannot be totally avoided.^4^ Testis is extremely sensitive to ionizing radiation,^5^ in which, as a finely tuned process, spermatogenesis is vulnerable to environmental toxicants.^6^ Thus, testis radiation protection is critical for radiation workers as well as doctors involved in radiology, radiotherapy and interventional therapy. Generally, ionizing radiation (IR) directly induces DNA damage in spermatogenic cells, which results in microenvironment change, cell degeneration and apoptosis. On the other hand, indirect effects induced by radiolysed water cause oxidative stress damage to testis.^7^, ^8^, ^9^ Male sperm density descending was obviously observed when exposed to 0.4 Gy ionizing radiation,^10^ and DNA damage and epigenetic DNA methylation changes can be also observed.^11^ Thus, exposure to IR is a high risk factor for male reproductive capacity impairment, alleviating radiation‐induced testis damage is a crucial problem to be solved.

Toll‐like receptors (TLRs) are kinds of pattern recognize receptors, which recognize conserved components of invading microbial pathogens and participate in innate and acquired immunity,^12^ In 2008, Burdelya et al^13^ firstly reported TLR5 agonist CBLB502 alleviated ionizing radiation‐induced tissue damage in vivo; since then, plenty of researches were carried out to investigate the protective effects of TLRs against ionizing radiation. Up to now, TLR2, TLR3, TLR4, TLR5 and TLR9 were demonstrated to play roles in ionizing radiation protection.^13^, ^14^, ^15^, ^16^, ^17^ Among which, TLR4 was found to be critical for the basal resistance to IR, and activation of TLR4 was proved to exert significant radioprotective effects. Monophosphoryl lipid A (MPLA) is a low‐toxicity TLR4 agonist and is proved to be 10 000 times less toxic than LPS (a classic TLR4 agonist). Our previous study demonstrated that in response to acute high dose radiation, MPLA effectively protected multiple organs, including intestine, spleen, marrow and testis.^18^ However, there are no relevant researches focusing on MPLA‐mediated radiation protection on specific radiosensitive organs or tissues, such as male testis, and which target cells upon TLR4 activation participate in testis radiation protection remains to be uncovered.

In the present study, we found that MPLA effectively alleviated radiation damage on testis in a TLR4‐dependent manner. Surprisingly, MPLA triggered the secretion of macrophage‐derived exosomes and these exosomes were accounted for the radioprotective effects. Exosomes are 30‐100 nm endosome‐derived vehicles,^19^ and they are produced by almost all kinds of eucaryote cells and distributed throughout body fluid environment. The complicated content, including functional protein, lipids, RNAs^20^, ^21^ grant exosomes playing important roles in cellular communication and signal mediation. Here, through a protein array, we identified exosomal G‐CSF and MDC as key components in testis radioprotection.

## 2 MATERIALS AND METHODS

### 2.1 Mice and treatment

Six‐week‐old male wild‐type C57BL/6 mice were obtained from Shanghai Ling Chang biological technology co., Ltd, and TLR4‐deficient mice and TRIF mutant mice were obtained from Model Animal Research Center of Nanjing University. Mice were kept in specific pathogen‐free (SPF) facility with sufficient sterilized mice food and water for all experiments. All the experiments associated with mice were approved by Second Military Medical University, China in accordance with the Guide for Care and Use of Laboratory Animals published by the US NIH (publication no. 96‐01). MPLA was bought from InvivoGen (Lot: MPL‐38‐02). For MPLA administration, mice were injected with diluted MPLA at dose of 50 µg/kg in 0.1 mL phosphate buffer saline 12 hours before irradiation through intragastric administration, whereas mice in control group were administrated with 0.1 mL phosphate buffer saline in the same way. After exposure to irradiation, mice were killed at different time‐points and subjected to related experiment as designed. For exosome inhibitor administration, GW4869 (Lot: S7609, Selleck, diluted in DMSO) was injected through intraperitoneal injection (2 µg/g) 2 hours prior to MPLA administration. Neutralizing antibodies were used to neutralize specific proteins. Specifically, mouse MIP‐2 antibody (MAB452, R&D); mouse MDC antibody (AF439, R&D); mouse RANTES antibody (AF478‐SP, R&D); and mouse G‐CSF (MAB414, R&D) were injected through intraperitoneal injection 2 hours prior to MPLA administration, and the dosage of antibodies refers to previous reports.^22^, ^23^, ^24^, ^25^

### 2.2 Cells and treatment

GC‐1 spg cells mice spermatogonias was purchased from BeNa Culture Collection; RAW264.7 was purchased from American Type Culture Collection. DMEM cell culture medium (for both RAW264.7 and GC‐1 spg cells) with 10% foetal bovine serum (Gibco) and 1% penicillin‐streptomycin solution (Hyclone) was obtained from PAA Laboratories. All cells were incubated in 37°C in a 5% CO_2_ humidified incubator. Cells were pre‐treated with MPLA at concentration of 1 µg/mL at 12 hours before irradiation and then, respectively, exposed to 0, 2, 4, 6 and 8 Gy irradiation and subjected to related experiments as designed. CHX (Sigma, Lot: C7698‐1G) was used to inhibit protein synthesis. Briefly, RAW264.7 was cultured for 24 hours to reach the confluence of 60%‐70%. Cell culture medium was replaced by CHX‐contained medium (1 µg/mL) at 12 hours before MPLA administration. At 12 hours after CHX pre‐treatment, the original cell culture medium was removed, and fresh cell culture, containing with MPLA (1 µg/mL) and CHX (1 µg/mL), was added in cell culture plate for the next 24 hours. This cell culture was used for protein‐free exosome extraction. For neutralizing antibody administration, GC‐1 spg cells was treated with respective neutralizing antibody together with exosome treatment. The neutralizing antibody was used at a concentration three times to the neutralizing dose (ND_50_).

### 2.3 Irradiation

60Co γ‐rays in Radiation Center (Faculty of Naval Medicine, Second Military Medical University, Shanghai, China) were used as irradiation generator. The average energy of ^60^Co‐γ rays is 1.25 MeV, and dose rate is 1 Gy/min. Mice and cells were exposed to different doses of radiation, depending on the requirements of experiments.

### 2.4 Testis cell cycle analysis

Six‐ to eight‐week‐old C57BL/6 male mice were killed 12 hours after 4 Gy irradiation, and then, testis from one side was isolated and grinded into cell suspension. Cell suspension was then centrifuged at speed of 800*g* and re‐suspended with PBS for three times. Next, cell suspension was stained with mixed dye solution (consists of 50 µg/mL propidium iodide [Transgene], 0.2% Triton X‐100 [Sangon Biotech] and 100 µg/mL RNAse‐A [Transgene]) for 15 min in 37°C. CytoFLEX (Beckman Coulter Company) was used for flow cytometry sample analysis.

### 2.5 Enzyme‐linked immunosorbent assay assay

C57BL/6 male mice were killed 21 days after 2Gy irradiation. Blood serum was isolated from blood drawn from angular vein venous before the animal was killed, and testis from one side was also isolated just after the animal was killed. Serum and testis homogenate were subjected to enzyme‐linked immunosorbent assay (ELISA) assay to determine testosterone level following the manufacturer's instructions (Westang Tech.).^26^

### 2.6 Sperm counting

To calculate epididymis sperm numbers, epididymis from one side was isolated and cut into tissue fragment in 2 mL 37°C normal saline. The sperm suspension was incubated for 10 minutes and then heated to 70°C in order to kill mice sperms. Sperms were counted by microscopic counting method.

### 2.7 Haematoxylin and eosin staining and TdT‐mediated dUTP nick‐end labelling staining

For haematoxylin and eosin (H&E) staining, mice were killed at day 1, day 7 after 4 Gy irradiation and at day 21 after 2 Gy irradiation. Testis from one side was isolated and fixed with 4% paraformaldehyde. Next, the samples were embedded in paraffin, cut into thin sections (4 μm thick) and stained with the H&E for the final histopathological studies. For TdT‐mediated dUTP nick‐end labelling (TUNEL) stain, mice were killed at 16 hours after 4 Gy irradiation. Testis from one side was made into tissue sections as mentioned above and subjected to TUNEL staining by using IF TUNEL kit (Roche, Lot: 11684817910) according to manufacturer's protocol.

### 2.8 Co‐culture system

The pore polycarbonate membrane (0.4 μm, 6.5 mm diameter) transwell chamber (product number: 3491; Corning Company) was used for the co‐culture system. In brief, 1*10^5^ RAW264.7 was seeded in transwell chambers, GC‐1 spg cells was cultured in the bottom of 24‐well plate, and transwell chambers and 24‐well plate were then combined according to manufacturer's instructions. For Western blot assay, 1.3*10^5^ GC‐1 spg cells were seeded in 24‐well plates, and for clonal formation assay, 100, 200, 400 and 800 GC‐1 spg cells were seeded, respectively, for 0, 2, 4 and 8 Gy irradiation. RAW264.7 in transwell chamber or GC‐1 spg cells in 24‐well plate was treated with MPLA 12 hours before irradiation. Transwell chambers were removed immediately after exposure to irradiation. GC‐1 spg cells in 24‐well plates were then subjected to clonal formation assay or Western blot assay.

### 2.9 Exosome purification and identification

The exosome purification kit (Umibio (Shanghai) Co., Ltd; Cat No: UR52101) was used for exosome extraction and purification. Briefly, RAW264.7 cell supernatants were isolated and centrifuged at 3000 ***g*** to remove cell debris. The supernatants were then mixed with exosome concentration solution in a 4:1 ratio and rested for at least 2 hours in 4°C. The mixture was then centrifuged at 10 000 ***g*** for 1 hour to separate exosome from cell culture. Next, exosome preliminary extraction was obtained by re‐suspending exosome precipitate with PBS. Finally, we obtained purified exosomes by centrifuge re‐suspended exosome at 3000 ***g*** for 10 minutes in exosome purification filter. ZetaView^®^ Nanoparticle Tracking Analyzer was used in exosome identification (Figure S1C).

### 2.10 Western blot assay

We obtained testis and cell protein samples by using M‐PER mammalian protein extraction reagent (#78501; THERMO) followed by manufacturer's instruction. DNA‐PKcs T2609 (Abcam; 1:1000), p‐ATR (Abcam; 1:1000), γH2AX (Abcam; 1:1000), TLR4 (Proteintech; 1:1000), Bax (Cell Signaling tech; 1:1000), Bcl2 (Cell Signaling tech.; 1:1000), caspase3 (Cell Signaling Technology; 1:1000), C‐caspase3 (Cell Signaling Technology; 1:1000) and β‐tubulin (Proteintech; 1:1000) were detected by Western blot assay, and the secondary antibody (1:5000) was purchased from Cell Signaling Technology.

### 2.11 Statistical analysis

Data were expressed as means ± the standard error of mean (SEM) for each experiment. The number of samples is indicated in the description of each experiment. We used an analysis of variance (ANOVA) followed by a Student‐Newman‐Keuls post hoc test for statistical analysis. Experiments for quantification were conducted in a blinded fashion, and all the experiments were repeated for at least 3 independent times.

## 3 RESULTS

### 3.1 MPLA alleviated IR‐induced injury in mice testis

**Figure 1:**
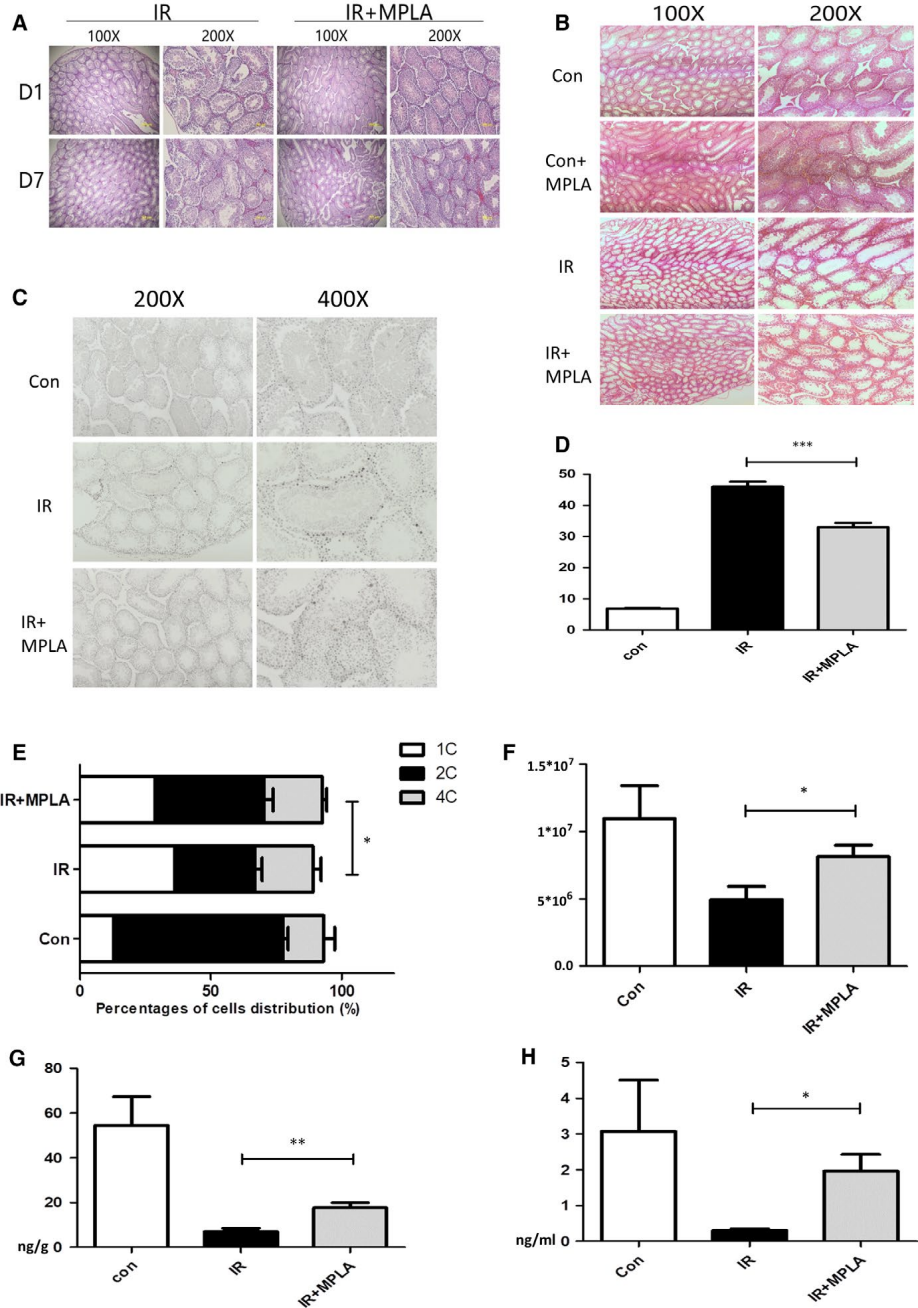
MPLA treatment before ionizing radiation (IR) alleviate IR injury in mice testis. A, On day 1 and day 7 after 4 Gy IR, testis was isolated and subjected to tissue sectioning and H&E staining. B, Mice were exposed to IR at dose of 2 Gy, at day 21 after irradiation; testis were isolated and subjected to tissue sectioning and H&E staining. C, Testis was isolated at 16 h after IR and fixed with polyformaldehyde; polyformaldehyde‐fixed paraffin‐embedded testis was stained with TUNEL method. D, TUNEL‐positive cells were counted in 200× field of view, ten fields of views were randomly selected in each group, and average numbers of TUNEL‐positive cells were calculated and showed. E, At day 21, sperms in epididymis were calculated by using microscope cell counting method. F, Flow cytometry was used to calculate 1c, 2c and 4c spermatogenic cells. G, H, Mice testosterone in testis and serum was examined by ELISA method 21 days after exposure to 2 Gy IR. Data were presented as mean ± SD (n = 3). *P < .05. **P < .01, ***P < .001

To determine the radioprotective effects of MPLA on testis, we administrated MPLA at the concentration of 50 µg/kg per mice by intragastric administration 12 hours before 2 Gy irradiation. On 16 hours, day 1, day 7 and day 21 after irradiation, testis was isolated and subjected to H&E staining or TUNEL staining. We found that testis of MPLA administration group maintained a relatively complete structure. Less necrotic cells and cavities in convoluted seminiferous tubules were found than that in IR group on 1, 7 and 21 days (Figure 1A,B). TUNEL staining showed that MPLA administration significantly decreased cell apoptosis after irradiation (Figure 1C,D). Next, we used flow cytometry to detect cell cycle distribution, and we found that MPLA remarkably increased 2C cell numbers 12 hours after irradiation compared to IR group (Figure 1E). This result indicated MPLA helps sustain more cells in normal cell cycle and reduce cells arrested in G2/M phase. To study the influence of MPLA on spermatogenic function, we collected blood serum and testis on 21 days after irradiation. We found that MPLA administration significantly maintained testosterone level in blood serum and testis compared to IR group (Figure 1G,H). Moreover, sperm number and motility were also significantly higher than that in IR group (Figure 1F). These results suggested that MPLA administration alleviates IR injury in mice testis and maintains testis normal function.

### 3.2 MPLA inhibited DNA damage and cell apoptosis in testis

**Figure 2:**
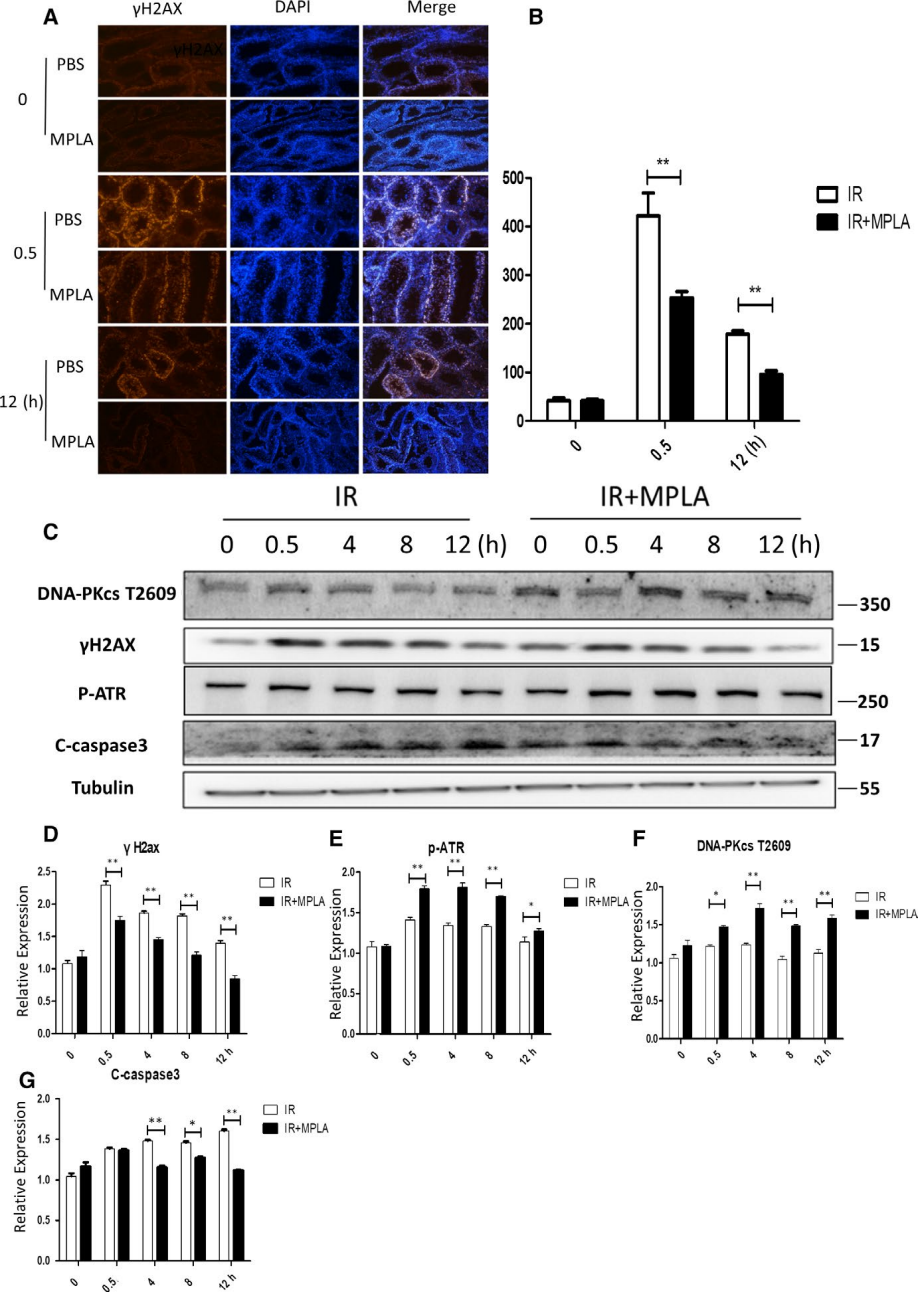
MPLA alleviated apoptosis pathway activation and helps activate DNA damage repair pathway. A, B, Immunofluorescence staining was used to determine γH2AX activation level in testis after IR, γH2AX positive cells were counted and calculated as showed in (B). (C)DNA‐PKcs t2609, γH2AX, p‐ATR, C‐caspase3 expression level was determined by western bolt assay, Tubulin served as a loading control. (D, E, F, G) Protein relative expression level was showed in (D‐G). D, Quantitative data of γH2AX. E, Quantative data of p‐ATR. F, Analysis of DNA‐PKcs T2056. G, Quantative analysis of c‐Caspase 3. Data were presented as mean± SD (n=3). *P < .05. **P < .01, ***P < .001

It is widely recognized that IR causes DNA strand breaks and subsequently induces cell apoptosis.^27^ Thus, we firstly examined γH2AX as an indicator of DNA double‐strand break^28^ at different time‐points by immunofluorescence method (Figure 2A,B). At 0.5 and 12 h after irradiation, numbers of γH2AX‐positive cells were much higher in IR group compared to MPLA administration group, which denoted that more DNA damage was repaired in MPLA‐treated group. Next, we used Western blot assay to detect changes in DNA damage response (DDR) and apoptosis signalling pathway (Figure 2C,D). We found that MPLA pre‐treatment remarkably increased the phosphorylation of DNA‐PKcs T2609 and ATR and decreased γH2AX level. We also examined cleaved‐caspase3 level as apoptosis pathway indicator and found less caspase3 was cleaved in MPLA‐treated group compared to IR group. Comprehensively, these results suggest that MPLA alleviated radiation‐induced DNA damage and cell apoptosis in testis.

### 3.3 MPLA protected mice testis through activating TLR4 on macrophages other than spermatogenia cells

**Figure 3:**
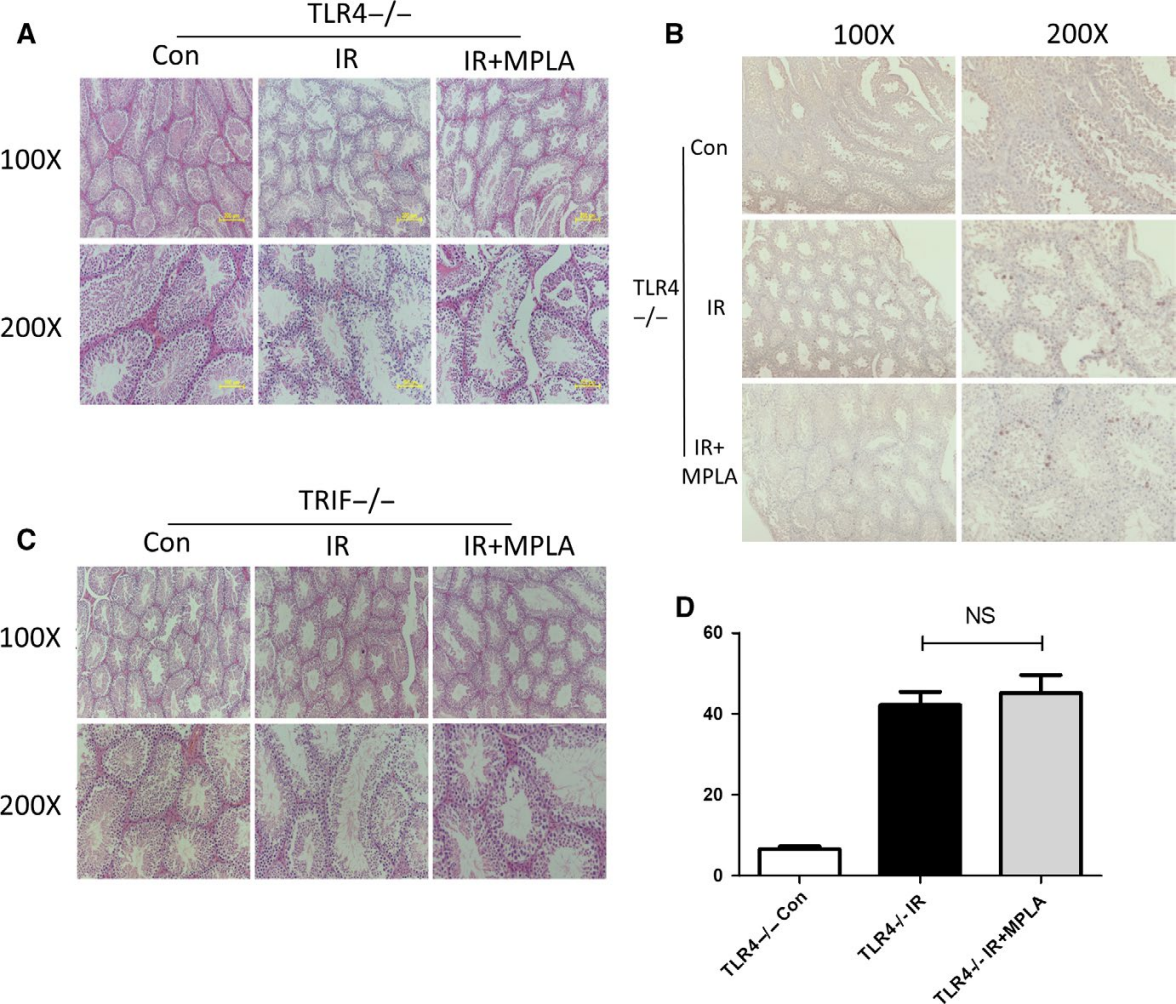
MPLA protected mice testis from IR injury via TLR4‐Trif–dependent pathway. A, Seven days after TLR4−/− mice exposed to 4Gy IR, mice testis was isolated and subjected to tissue sectioning and H&E staining. B, TUNEL method was used to determine TLR4−/− mice cell apoptosis in testis 16 h after exposure to IR. C, TUNEL‐positive cells were counted in 200X field of views, 10 fields of views were randomly selected in each group, and average numbers of TUNEL‐positive cells were calculated and showed. D, Seven days after TRIF−/− mice exposed to 4 Gy IR, mice testis was isolated and subjected to tissue sectioning and H&E staining. Data were presented as mean ± SD (n = 3). *P < .05. **P < .01, ***P < .001

Next, we explored whether radiation protection effects of MPLA on testis are TLR4‐dependent. We isolated testis form TLR4‐deficient mice 16 hours and 7 days after irradiation and subjected tissue samples, respectively, to H&E staining (Figure 3A) and TUNEL staining (Figure 3B,C). We found no significant difference in cell apoptosis or testis damage degree between MPLA administration group and IR group. TRIF is reported to be one of the adaptors downstream of TLR4, and MPLA was reported as a TRIF‐biased TLR4 agonist.^29^ By using TRIF‐deficient mice, we confirmed that MPLA produced radiation protection effects through TLR4‐TRIF pathway (Figure 3D), because testis damage degree showed no difference with or without MPLA treatment in TRIF‐deficient mice.

**Figure 4:**
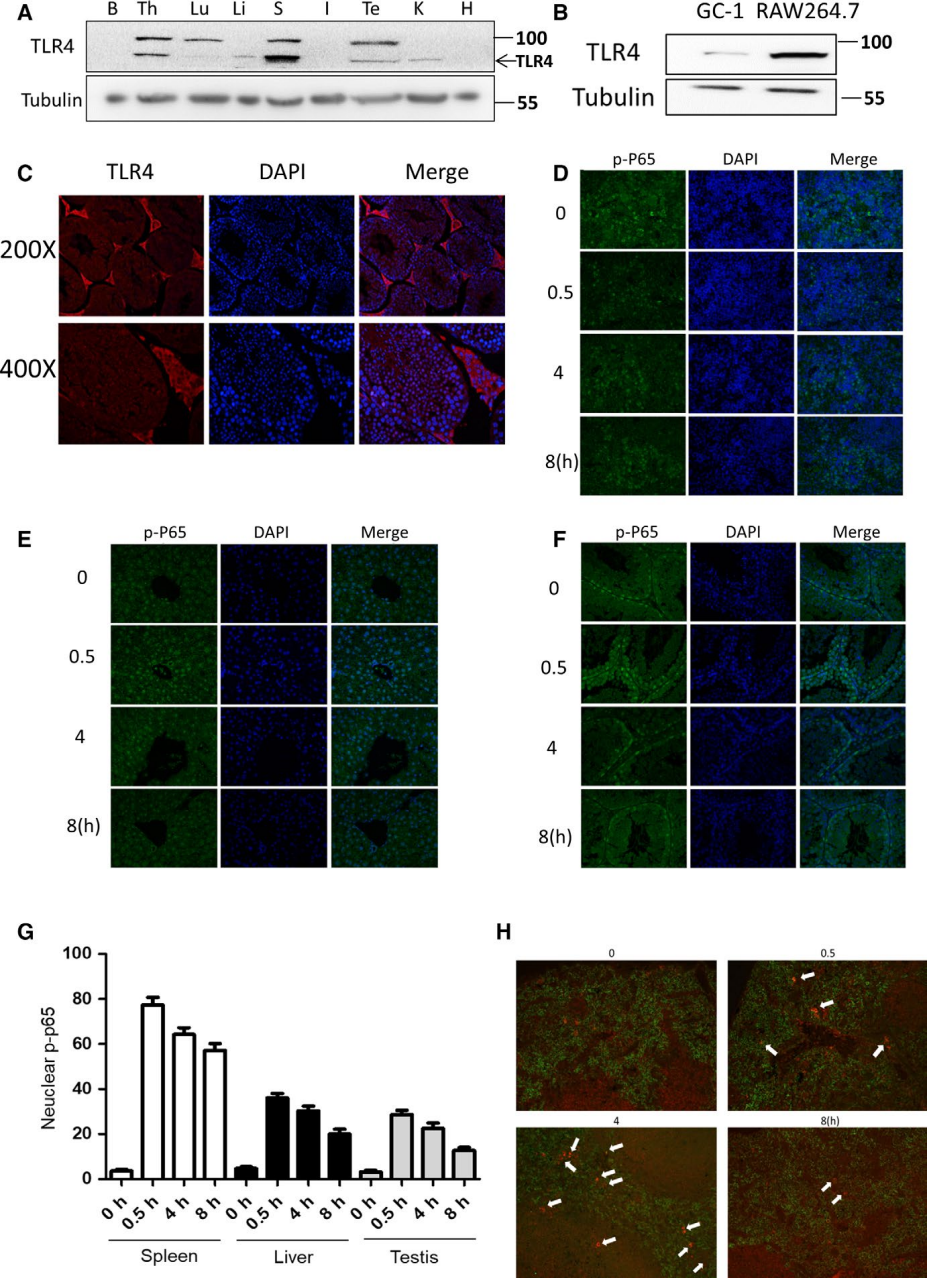
The distribution of TLR4 and the activation level of TLR4‐NF‐κB pathway in different tissues. A, TLR4 expression level in different organs was examined by Western blot assay, B represents brain, TH represents thymus, Lu represents lung, Li represents liver, S represents spleen, I represents intestine, TE represents testis, K represents kidney, and H represents heart. B, TLR4 expression level in GC‐1 and RAW264.7 was examined by Western blot assay. C, Immunofluorescence method was used to determine TLR4 expression level and distribution in testis. D‐F, Mice were treated with MPLA at dose of 50 µg/kg; spleen (D), liver (E) and testis (F) were isolated at different time‐point and then were subjected to tissue sectioning; and p‐P65 was stained by immunofluorescence method. G, Ten 200× field of views were randomly selected, and cells with p‐P65 nuclear translocation were calculated and showed in G. H, After 50 µg/kg MPLA administration, mice testis was isolated at different time‐point. p‐P65 and F4/80 immunofluorescence co‐staining was used to determine TLR4 activation level in mice macrophages

Deriving from spermatogonial stem cells, spermatogonias differentiate into primary spermatocytes and eventually into sperms; on the other hand, spermatogonias also maintain stable numbers through self‐renew.^30^ Surprisingly, although testis TLR4 expression was detected by Western blot assay (Figure 4A), almost no TLR4 expression in spermatogonias can be detected (Figure 4B,C). Moreover, no radiation protection effects were detected by clonal formation assay or Western blot assay when treated GC‐1 directly with MPLA (Figure S2A‐C). To figure out which target cells of TLR4 were accounting for the radioprotective effect of MPLA, we performed a NF‐κB staining assay in multiple tissues. NF‐κB is reported as TLR4 downstream adaptor and consists of five subunits including p65. Once activated, p65 would be phosphorylated at Ser536 and translocated into nuclear.^31^ As showed in Figure 4D‐G, p‐65 nuclear translocation in spleen was significantly higher than that in testis or liver. Furthermore, we stained phosphor‐p65 and F4/80^32^ by co‐immunofluorescence method (Figure 4H) and found that large amount of macrophages in spleen was activated. Taken together, our data showed that MPLA possibly protected testis against IR through TLR4 pathway activation on macrophage instead of activating TLR4 in spermatogonias directly.

### 3.4 Macrophage‐derived exosomes were critical for the radioprotective effects of MPLA

**Figure 5:**
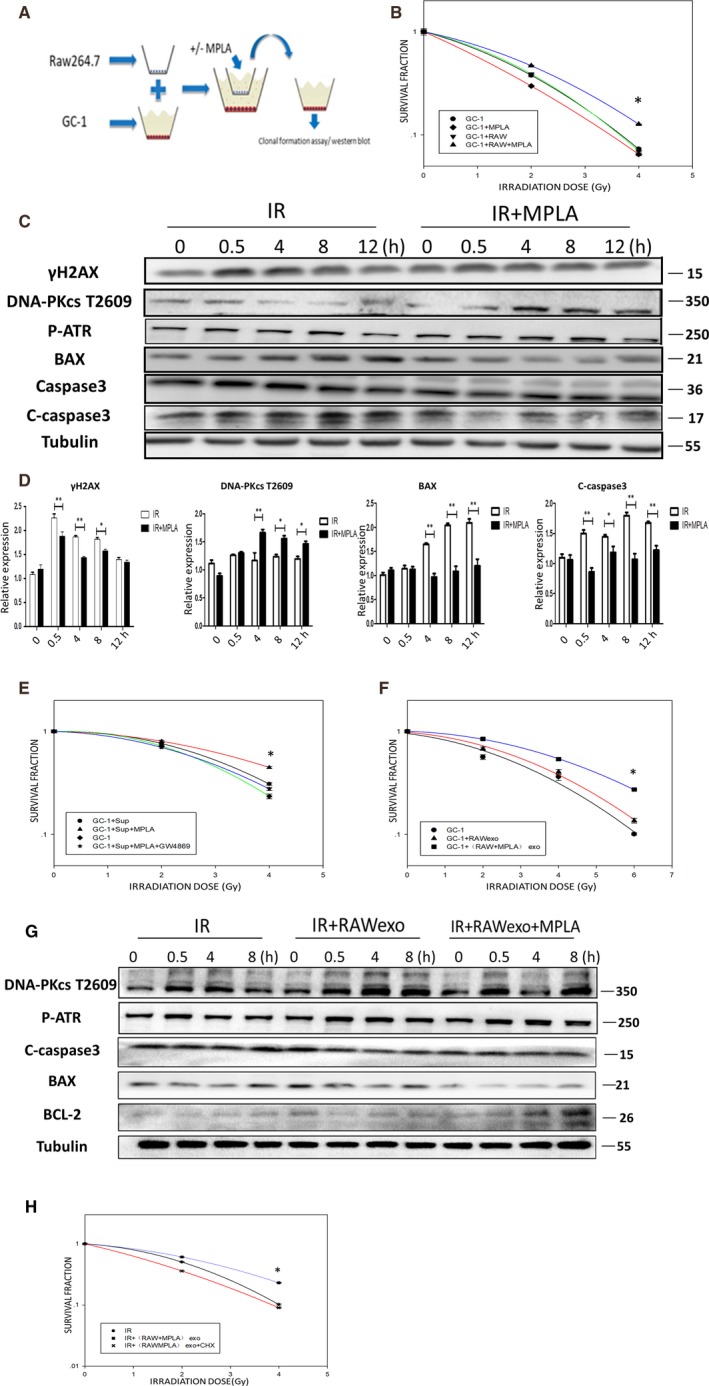
MPLA produced IR protection effects on spermatogonias depending on RAW264.7 TLR4 activation and RAW264.7‐derived exosomes. A, Schematic of co‐culture system. B, Clonal formation assay was used to determine proliferative capacity of GC‐1 cells in co‐culture system after IR. C, The GC‐1 cells in co‐culture system were treated with or without MPLA. Expression level of γH2AX, DNA‐PKcs t2609, p‐ATR, Bax and C‐caspase3 was examined by Western blot assay. D, Protein relative expression level was calculated as showed. E, Proliferative capacity of GC‐1 was examined by clonal formation assay after RAW264.7 supernatant treatment. F, Proliferative capacity of GC‐1 was examined after RAW264.7‐derived exosomes treatment. G, Expression level of DNA‐PKcs t2609, p‐ATR, Bax, C‐caspase3 and Bcl2 was examined by Western blot assay after RAW264.7‐derived exosomes treatment. H, Proliferative capacity of GC‐1 was tested by clonal formation assay after RAW264.7‐derived exosomes treatment with or without CHX. Data were presented as mean ± SD (n = 3). *P < .05. **P < .01, ***P < .001

Consistent to our in vivo data, MPLA showed little protective effect on murine spermatogonia GC‐1 cell lines, which showed low TLR4 expression level (Figure S2A‐C). Next, we explored whether macrophages, as highly activated cells by MPLA, can promote spermatogonias survival and alleviate damage after irradiation. We established RAW264.7 and GC‐1 co‐culture system as illustrated in Figure 5A. This co‐culture system was pre‐treated with MPLA at 12h before irradiation, and after irradiation, GC‐1 was subjected to clonal formation assay or Western blot assay. As showed in Figure 5B, either treated with MPLA or co‐cultured with RAW264.7 did not produce radiation protection effects on GC‐1, but co‐cultured with RAW264.7 and together with MPLA treatment showed significant alleviation to cell death after irradiation. We examined DDR pathway as well as apoptosis pathway (Figure 5C,D) and found that DNA‐PKcs T2609 and p‐ATR showed higher activation level, whereas γH2AX, Bax and cleaved‐caspase3 levels were reduced in co‐cultured GC‐1 group with MPLA treatment. These results indicated that MPLA treatment with RAW264.7 co‐culture system alleviated DNA damage and cell apoptosis in GC‐1 cells. The 0.4um pore polycarbonate membrane in co‐culture system can completely prevent cell penetration; thus, we excluded direct cell contact and focused on RAW264.7‐derived excretion as the key factor in this indirect radiation protection effect. By using supernatant transfer method together with clonal formation assay, we found that MPLA‐stimulated RAW264.7 supernatant promoted GC‐1 survival after irradiation. Importantly, when pre‐treated RAW264.7 with GW4869, a widely recognized exosomes inhibitor, MPLA‐stimulated RAW264.7 supernatant showed obviously defected protection effect on GC‐1 (Figures 5E and S3B). This result suggested that exosomes may be the key factor for radiation protection effects. In the next step, we treated GC‐1 directly with exosomes extracted from RAW264.7 supernatant, and cells were then subjected to clonal formation assay and Western blot assay. Interestingly, MPLA‐treated RAW264.7‐derived exosomes showed higher survival promotion effect on GC‐1 compared to MPLA‐untreated RAW264.7 group or IR group (Figure 5F). Moreover, pro‐apoptotic pathway was less activated and DDR pathway was activated in MPLA‐RAW264.7‐exosome group compared to others (Figure 5G). Taken together, we demonstrated that MPLA produced radioprotective effects on spermatogonias via TLR4‐dependent macrophage exosome secretion.

### 3.5 Macrophage‐derived exosomal G‐CSF and MIP‐2 stimulated by MPLA are dispensable for the radioprotective effects on testis

**Figure 6:**
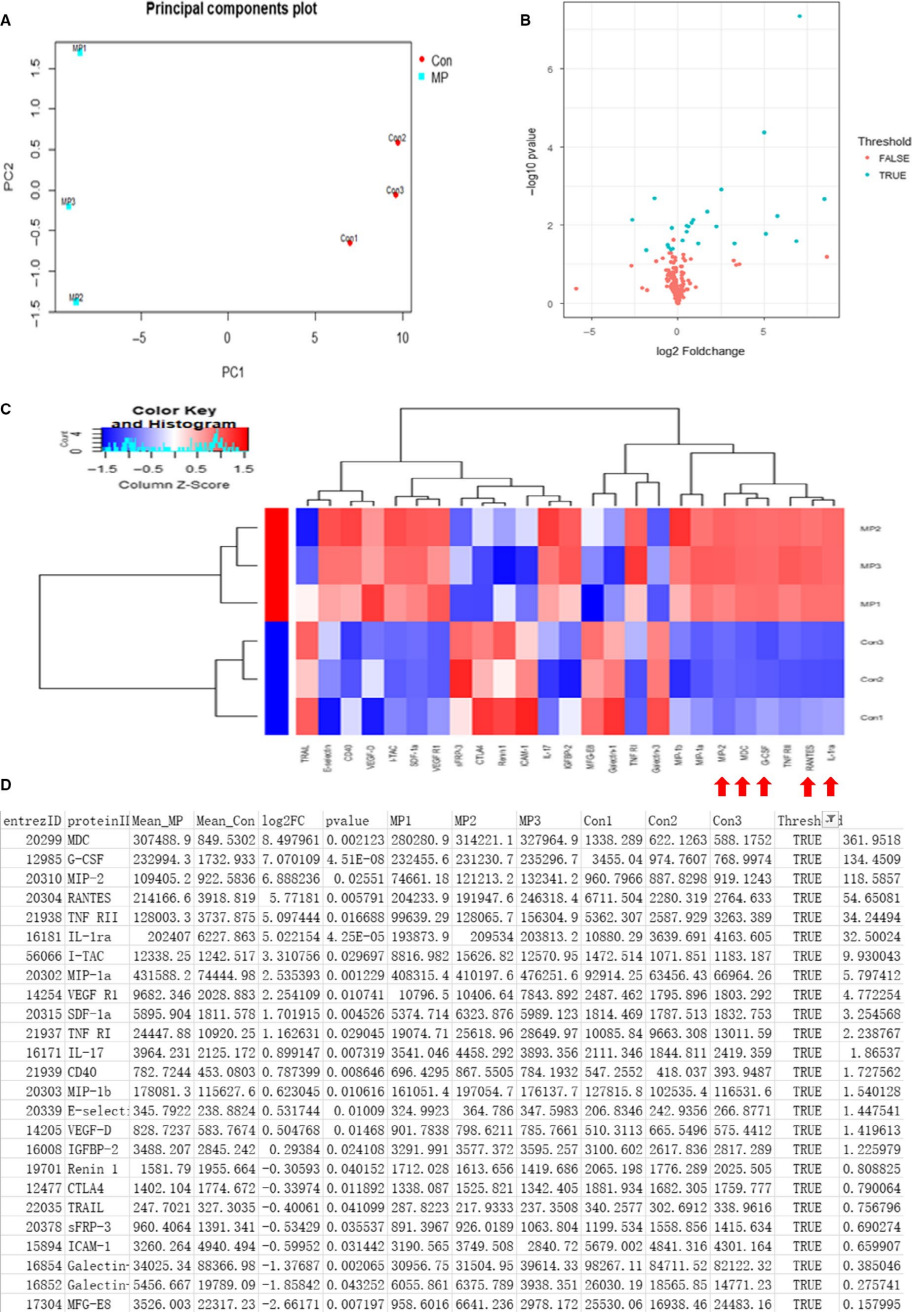
The protein expression profile from RAW264.7‐derived exosomes with or without MPLA treatment. A, The result of principle components analysis. B, Cluster heat map result. C, Heat map result. D, The list of proteins with significant expression level changes

To identify the key factor inside exosomes involved in radiation protection, we firstly used CHX to block the protein translation and found that after CHX treatment, the radioprotective effects of MPLA were abrogated (Figure 5H). Then, we used protein array to explore the specific protein which produced radiation protection effects in RAW264.7‐derived exosomes. We firstly confirmed that MPLA treatment significantly changed the protein expression profile as suggested in principle component analysis (Figure 6A). Next, 25 proteins with significant changes in expression levels were identified (Figure 6B), including 17 proteins with up‐regulated expression level and 8 proteins with down‐regulated expression level (Figure 6C). As showed in Figure 6D, many cytokines and chemokines were elevated in a great extent, and we focused on the proteins changes more than 10‐fold.

**Figure 7:**
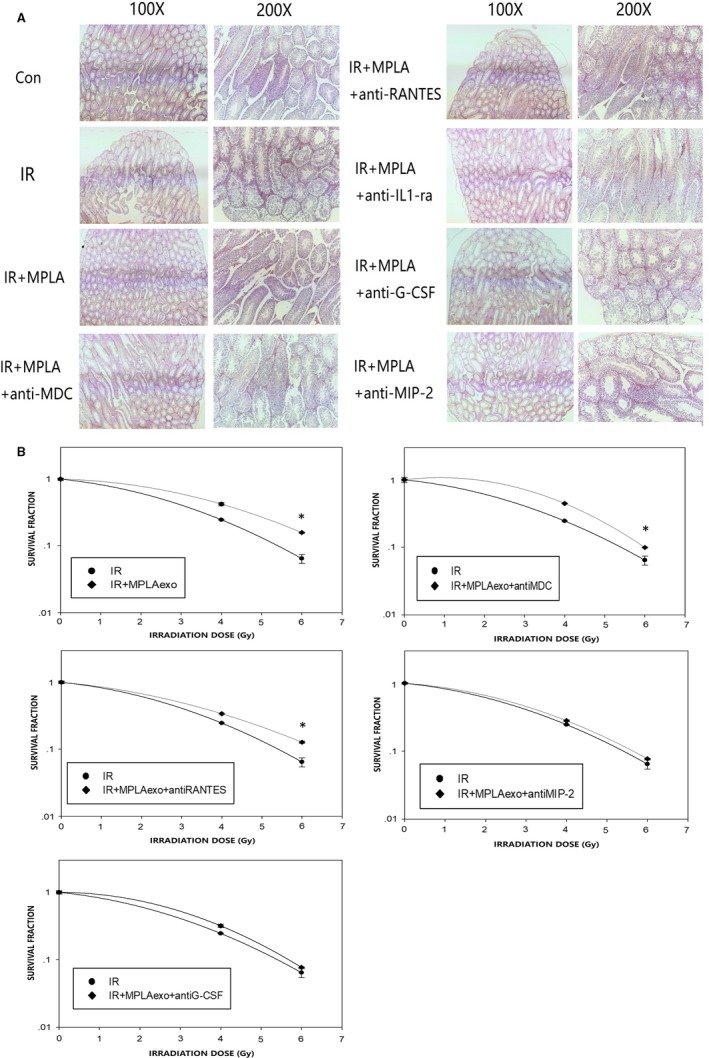
G‐CSF and MIP‐2 in exosomes were the main protection effectors in testis radiation protection. A, On day 7 after neutralizing antibody administration and 4 Gy irradiation, mice testis was isolated and subjected to H&E staining. B, Clonal formation assay was used to determine GC‐1 cell viability after exosomes treatment and irradiation exposure with or without neutralizing antibody. Data were presented as mean ± SD (n = 3). *P < .05. **P < .01, ***P < .001

**Figure 8:**
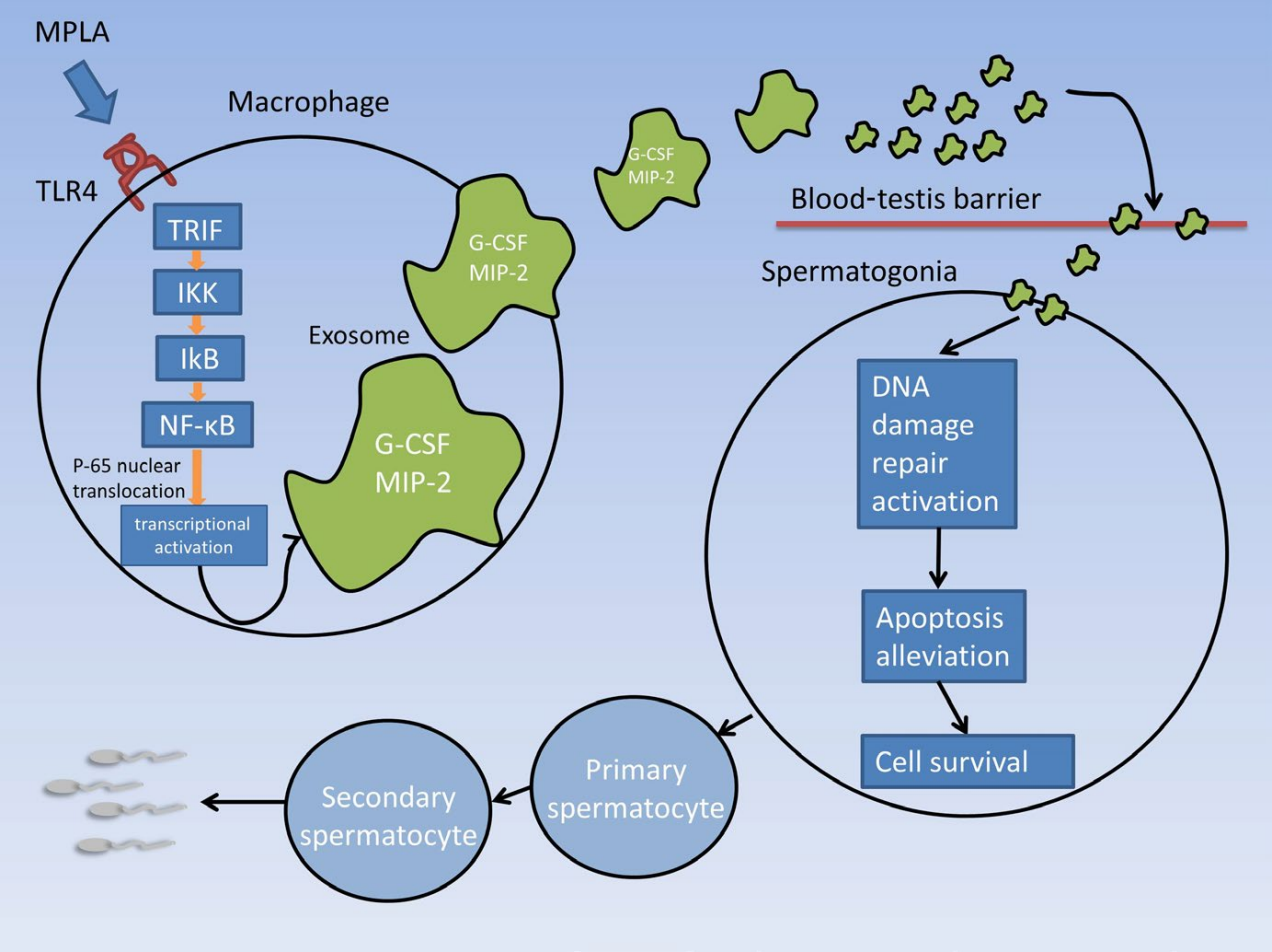
Proposed model for MPLA produced testis radiation protection. In macrophages, TLR4‐TRIF pathway was activated after MPLA administration. Subsequently, NF‐κB subunit p‐65 was phosphorylated and translocated into nuclear, which resulted in expression of multiple cytokines and chemokines. G‐CSF and MIP‐2, as the important mediators in testis radiation protection, were transported by macrophage‐derived exosomes and across the blood‐testis barrier and eventually promoted spermatogonias survival after irradiation

Among all, we selected four proteins with the most obvious changes in expression and used neutralizing antibody to validate their respective role in radiation protection both in vivo and in vitro. In histological examination, we observed that the protective effects of MPLA were inhibited in mice treated with G‐CSF neutralizing antibody and MIP2 neutralizing antibody (Figure 7A). We further validated the radiation protection effect in vitro by using clonal formation assay, we found that using G‐CSF neutralizing antibody or MIP‐2 neutralizing antibody significant counteracted radiation protection effects of RAW264.7‐derived exosomes (Figure 7B). Taken together, we detected that G‐CSF and MIP‐2 from MPLA‐stimulated RAW264.7‐derived exosomes were the main two contributors against radiation‐induced testis damage. In this study, we found that TLR4 contributes to testis radioprotection through exosomal factors, which provide novel mechanism (Figure 8).

## 4 DISCUSSION

To our knowledge, this was the first study that demonstrated TLR4 agonist MPLA‐stimulated macrophage‐derived exosomes participate in testis radiation protection. We detected that MPLA alleviated damage of testis and spermatogonias induced by IR, and maintained testis function by stimulating mice macrophage secreting G‐CSF– and MIP‐2–enriched exosomes. In in vivo study, we observed that MPLA treatment obviously alleviated testis structure damage both in short‐term (7 days) and long‐term (21 days). Consistently, testis endocrine function and sperm generation function also were maintained well in MPLA‐treated group. However, spermatogonias in testis express trace TLR4 as detected by testis sanction immunofluorescence staining and Western blot assay. Moreover, we observed significant TLR4 activation in spleen macrophages after MPLA administration, this give us an idea that distant cell/organ‐derived secretion might play roles in testis radiation protection effects. By using exosome inhibitor GW4869 in vitro, we preliminary confirmed that macrophage‐derived exosomes were accounted for testis radiation protection. Specifically, G‐CSF and MIP‐2 in macrophage‐derived exosomes were determined to be the core component in testis radiation protection as their neutralizing antibodies abrogated the radioprotective effects of MPLA.

It is widely accepted that IR‐induced DSBs are the most important reason for cytotoxic lesion. DSBs can be induced by either IR direct damage or IR‐induced reactive oxygen species (ROS). Non‐homologous end joining (NHEJ) or homologous recombination (HR) are the two main repair methods. As NHEJ repair does not require a template and occurs in all stages of the cell cycle, NHEJ is the primary repair method in mammals.^33^ DNA‐PKcs is an important component for KU70/80/DNA‐PKcs complex in NHEJ repairing process, which bound to DNA damage site, phosphorylate downstream protein and eventually mediate DNA damage repair. Moreover, DNA‐PKcs is reported often to be phosphorylated at T2609 phosphorylation cluster, and mutant studies on DNA‐PKcs defective at the T2609 cluster indicated that this phosphorylation is important for DSBs repair and genetic stability.^34^ On the other hand, ATR is also reported as an important component in DNA damage repair and cell cycle checkpoint. We found that MPLA administration in vivo obviously enhanced the activation of DNA‐PKcs T2609 and p‐ATR, which indicated a higher DNA repair activity. However, in in vitro study, the untreated RAW264.7‐derived exosomes also showed certain activation ability on GC‐1 DDR pathway. These results suggested that macrophage‐derived exosomes may play roles in maintaining genomic stability or cell self‐renewal regulation under normal conditions.

Apoptosis is a common way of programmed cell death when DNA damage cannot be repaired. DNA damage most often activates the extrinsic death receptor apoptosis pathway and/or the intrinsic mitochondrial apoptosis pathway. The activation of mitochondrial apoptosis pathway depends on the release of cytochrome c from the mitochondria into the cytoplasm, which is governed by pro‐apoptotic proteins, such as Bax (Bcl‐2–associated X protein) and Bak (Bcl‐2 homologous antagonist/killer), and anti‐apoptotic proteins such as Bcl‐2 (B‐cell lymphoma 2) and Bcl‐XL (B‐cell lymphoma‐extra large).^35^ In addition, caspase3 is the common downstream molecule of various apoptotic pathways and is considered to be a major performer of the apoptotic process.^36^ In the present study, we examined BAX, BCL‐2 and cleaved‐caspase3 as apoptosis pathway indicator and identified significant anti‐apoptosis effects on mice testis and spermatogonias after MPLA administration both in vivo and in vitro. Taken together, we believe that the MPLA could exert radiation protection effect on testis by enhancing DNA damage repair activity and inhibiting apoptosis caused by DNA damage.

Macrophages are derived from monocytes and participate in innate immunity, and the ability to secrete cytokines and chemokines is an important basis for macrophages in immune response and antigen presentation.^37^ The TLR4‐mediated reaction to inflammatory stimulation of macrophage is a highly complex cross‐talk pathway. Once stimulated, TLR4 receptor complex activates its downstream pathway via recruitment of adaptor proteins, mainly including myeloid differentiation factor 88 (MyD88) and TIR‐containing adapter molecule (TRIF, also known as TICAM‐1). Although in early study, TRIF‐dependent pathway was regarded mainly participating in IFN‐gamma production, recent study proved that both MyD88‐ and TRIF‐dependent pathways are involved in cytokine and chemokine production.^38^, ^39^ Actually, the important role of TRIF in TLR4 singling pathway has been reported by Mata‐Haro et al, in which they found that MPLA is a TRIF‐biased agonist of TLR4 as a vaccine adjuvant. And in fact, we observed that MPLA showed defected protection effects on testis in TRIF‐deficient mice, which also demonstrated that MPLA produced radiation protection effect on testis is TLE4‐TRIF pathway biased.

It is widely reported that MPLA stimulates immune cells secreting plenty of pro‐inflammatory cytokines and chemokines, including IL‐1β, IL‐6, and TNF‐α.^40^ Interestingly, according to our study, there were significant changes in the expression levels of 25 proteins in MPLA‐stimulated macrophage‐derived exosomes, among which, the expression level of MDC, G‐CSF, MIP‐2, RANTES, TNF‐RII and IL‐1ra increased by tens or even hundreds of times. This is the first time that we identified such a distinct protein expressing characteristics between cell supernatant and exosomes after MPLA treatment, which suggested that the regulation of protein secretion in exosomes was distinct from directly secreted proteins. Moreover, we detected obvious NF‐κB pathway activation in macrophages after MPLA treatment (Figure S3A), and we deduced that the different protein expression character between exosome proteins and directly secreted proteins formed after NF‐κB nuclear translocation and in the process of transcription or post‐transcriptional regulation, which worth to be further explored.

In a study conducted by Antonio Hernandez,^38^ G‐CSF and MIP‐2 (CXCL‐2) were found to be important in neutrophils mobilization and recruitment after MPLA treatment, which suggested their potential roles in MPLA response. Moreover, G‐CSF has already been reported to protect spermatogenesis after alkylating chemotherapy.^41^, ^42^ Here, we firstly demonstrated that G‐CSF and MIP‐2 were the two key proteins in exosomes to produce testis radiation protection effect, which may help renew our knowledge of these two molecules in the radiation protection. However, there are no prior published studies defining the contributions of MIP‐2 in testis radiation protection, and its potential roles in anti‐apoptotic or DNA damage repair activation should also be explored.

Exosomes are widely studied as cellular communication mediator, and the lipid surface structure grants exosomes to be potential drug carriers in delivering drugs into blood‐brain barrier (BBB) or blood‐testis barrier. It has been demonstrated that cell‐derived exosomes can cross the BBB model under stroke‐like conditions in vitro,^43^ and it is reported that exosomes could be potentially used as a carrier for anti‐cancer drugs in brain delivery in brain cancer treatment.^44^ Although the basic structure of BBB and blood‐testis barrier remains some differences, we believe that exosomes could enter the blood‐testis barrier by endocytosis like entering the blood‐brain barrier.^45^ However, the direct evidence for exosomes entering testis should be confirmed in further studies.

We believe that MPLA has great advantageous over other TLR4 agonists and is promising in clinical application as radiation protection drug on male reproductive system. MPLA is a low‐toxicity TLR4 agonist with TLR4 activation effect. According to our experimental results, MPLA produced significant testis radiation protection effects at the dosage of 50 mg/g, and there were no obvious side effects at the dosage 250 mg/g at least. This result suggested a wide range of safety dosage in clinical administration. It is reported that MPLA stimulates less pro‐inflammatory factors than LPS or other TLR4 agonists in endotoxin shock model or in macrophages, including IL‐6, IL‐12p40, TNF‐α and IL‐1β,^46^ and its reduced toxicity may be in part caused by its inability to activate certain types of immune cells, such as human circulation monocytes and mast cells.^40^ For now, MPLA has been used as immunoadjuvant in the field of vaccine development for various diseases, such as cancer, malaria, hepatitis B and pollen allergy, some kinds of vaccines have been approved by FDA or in the stage of Phase III clinical trial, and more than 300 000 people have received anti‐cancer vaccine injections containing MPLA.^47^ These large‐scale clinical application studies have fully demonstrated the reliable safety and the potent immune activation efficacy of MPLA and will provide important references for its radiation protection use. It is also recognized that MPLA could enhance the body immune response to outside invaders through enhancing antigen presentation and regulating Th1/Th2, etc, and these effects may help in defending infectious after irradiation, which are meaningful for irradiated patients in clinical treatment.

Taken together, we firstly demonstrated that MPLA helped alleviate testis radiation injury. We found that this effect is mediated by MPLA‐stimulated macrophage‐derived exosomes. Furthermore, we identified G‐CSF and MIP‐2 were the two specific exosomes proteins participating in testis injury alleviation. These new findings will help us better understand the role of TLR4 in radiation protection and give us new thought on exosomes‐mediated protection against ionizing radiation injury.

## CONFLICT OF INTEREST

The authors have no conflicts of interest to disclose.

## AUTHOR CONTRIBUTION

FG, XS and JC contributed to study concept and design, carried out experiments, prepared the manuscript and obtained funding. Z Liu, KC, Z Liao and YY: carried out experiments, data analysis and figure preparation. QW, CL, KC and YC: carried out experiments. XL: contributed to study design and obtained funding.

## Supporting information

FigS1

FigS2

FigS3

## Data Availability

All data are available upon request.
