# Supplementary figures and images for: Monophosphoryl lipid A alleviated radiation‐induced testicular injury through TLR4‐dependent exosomes
Source: J Cell Mol Med. 2020 Mar 5;24(7):3917–30. doi: 10.1111/jcmm.14978 (PMC7171420; doi:10.1111/jcmm.14978)

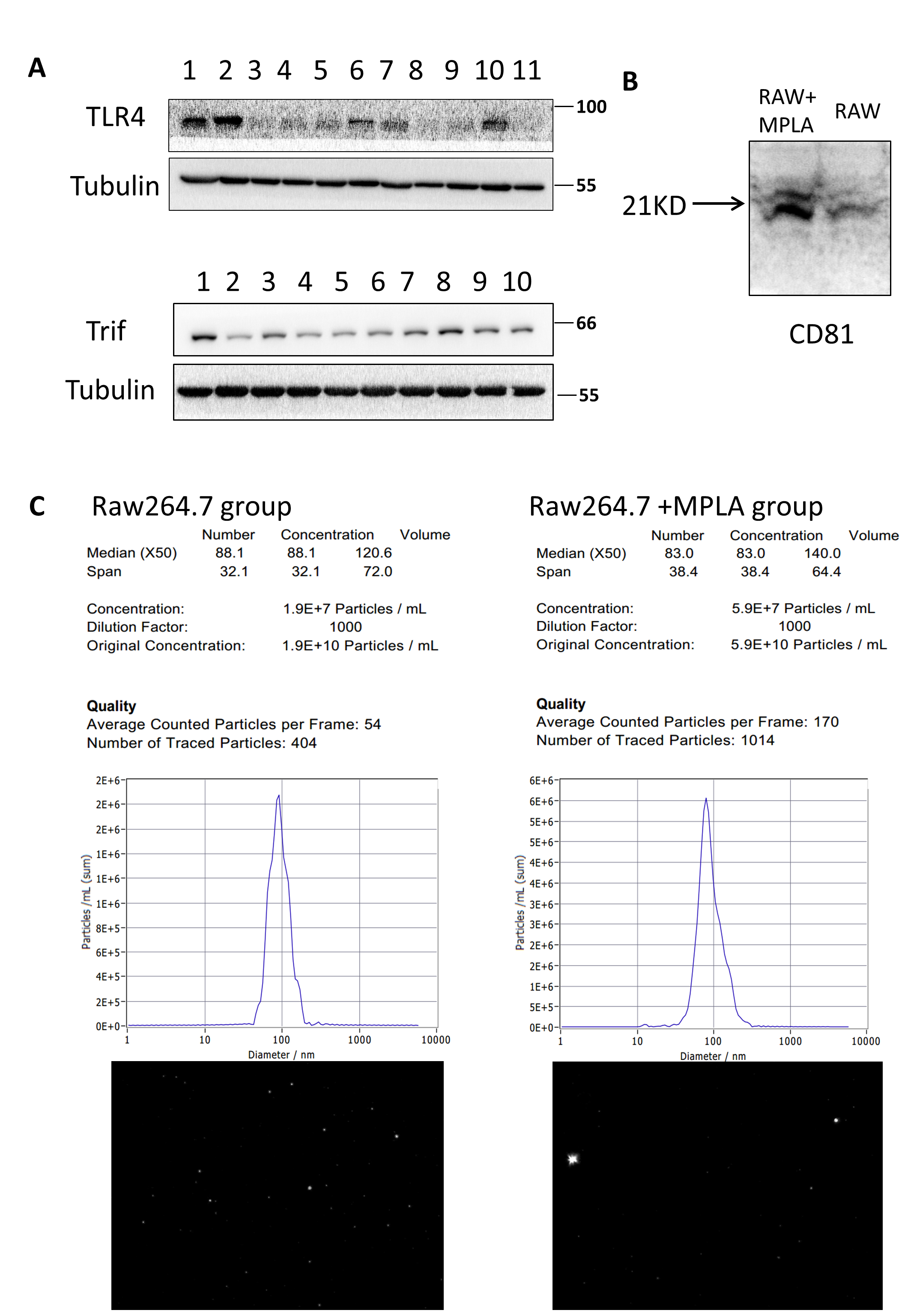

Supplement: Supplementary file 1 — FigS1 [file JCMM-24-3917-s001.TIF]

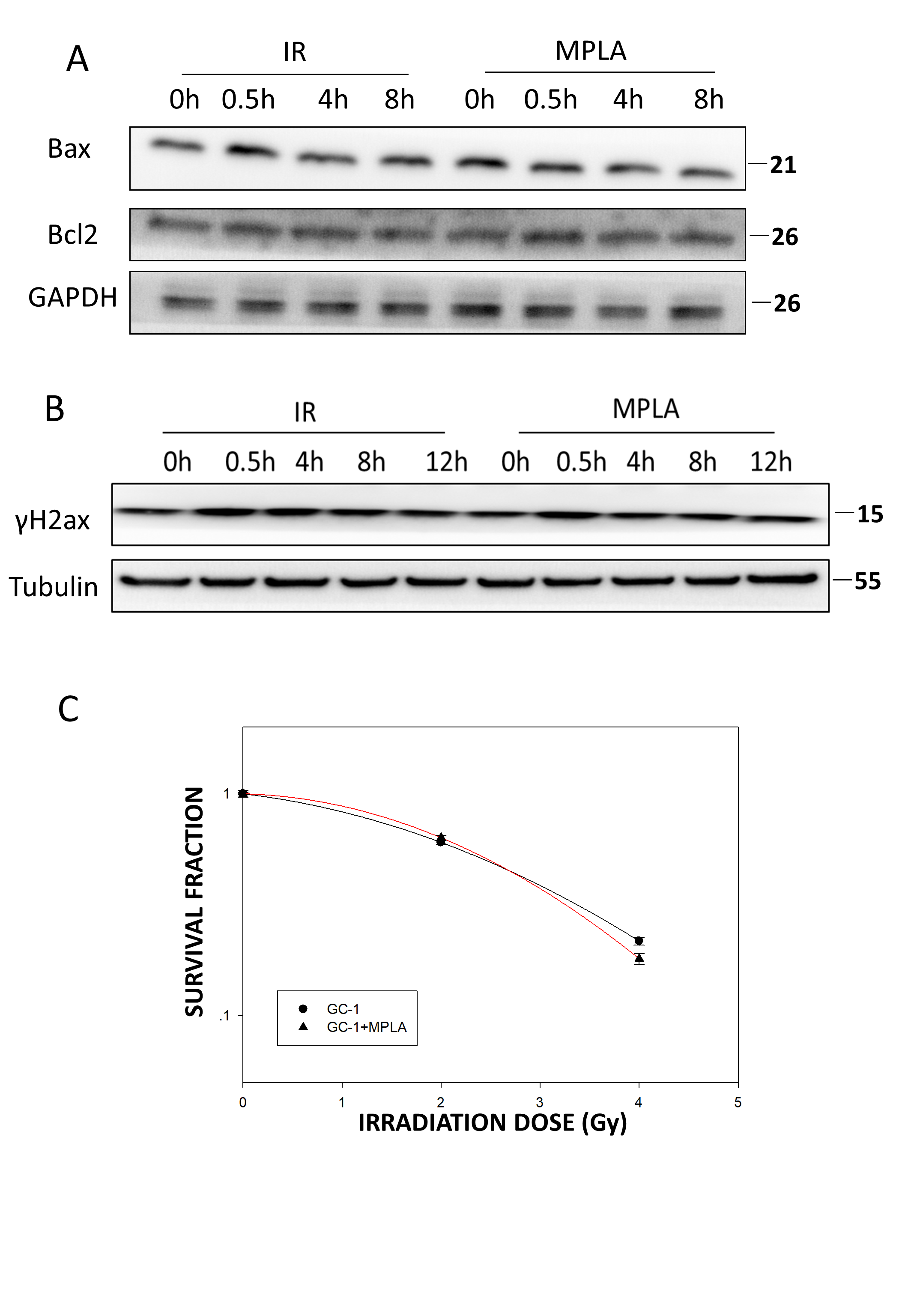

Supplement: Supplementary file 2 — FigS2 [file JCMM-24-3917-s002.TIF]

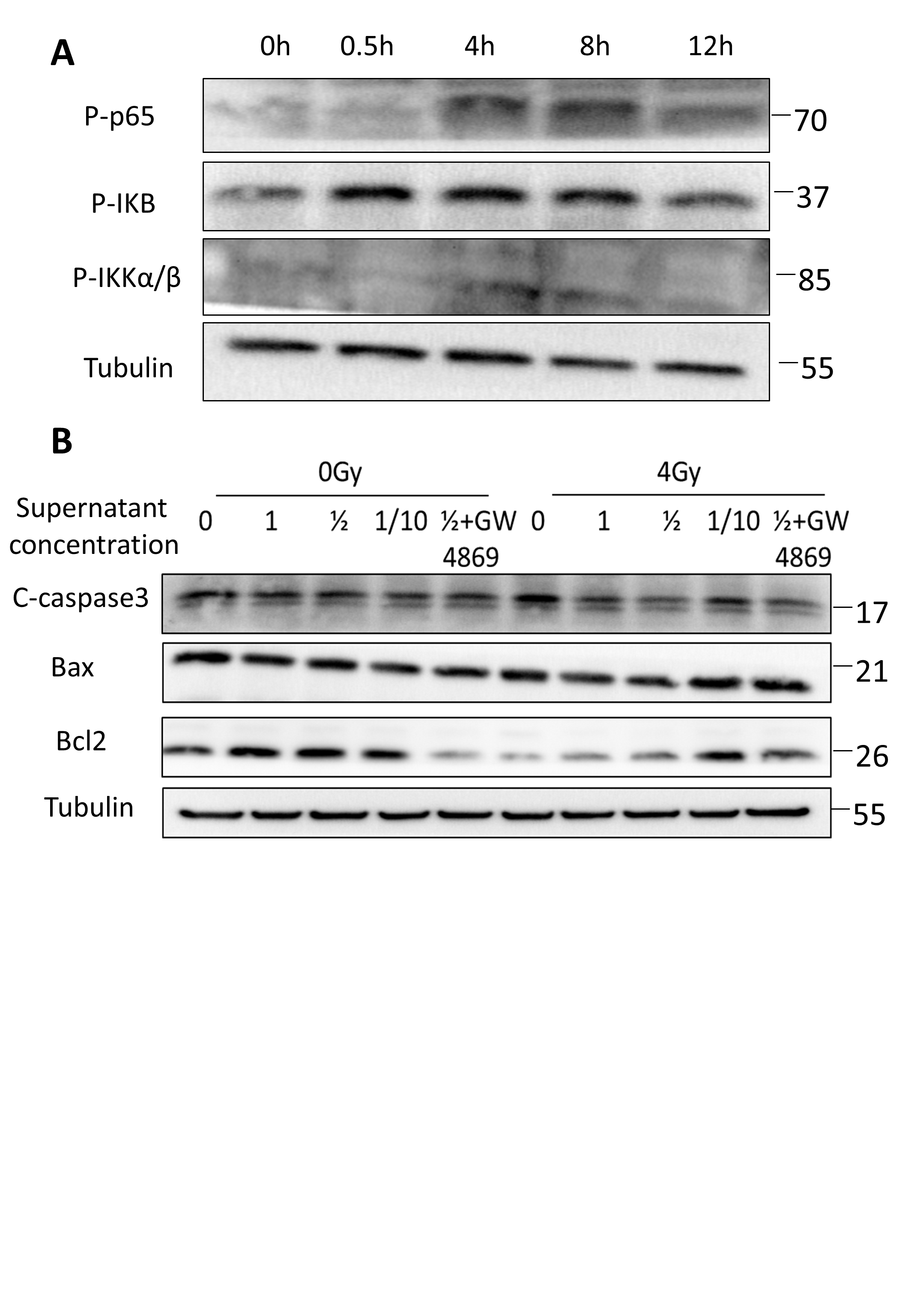

Supplement: Supplementary file 3 — FigS3 [file JCMM-24-3917-s003.TIF]
